# White and Red Sorghum as Primary Carbohydrate Sources in Extruded Diets of Felines

**DOI:** 10.3389/fvets.2021.668255

**Published:** 2021-04-20

**Authors:** Patrick von Schaumburg, Fei He, Sandra L. Rodriguez-Zas, Bruce R. Southey, C. M. Parsons, Maria R. C. de Godoy

**Affiliations:** ^1^Department of Animal Sciences, University of Illinois at Urbana-Champaign, Urbana, IL, United States; ^2^Division of Nutritional Sciences, University of Illinois at Urbana-Champaign, Urbana, IL, United States

**Keywords:** carbohydrate, felines, gut health, microbiota, sorghum, nutrient digestibility, ancient grain

## Abstract

The research objectives were to evaluate the effect of dietary supplementation of white (**WSH**) and red (**RSH**) sorghum grains on gastrointestinal health of felines through the determination of apparent total tract macronutrient digestibility (**ATTD**), fecal characteristics, fermentative end-products, and microbiota, compared with a traditional corn-based diet. We hypothesize that inclusion of RSH and WSH, respectively, would be well-accepted by cats, and the RSH and WSH diets would be comparable to corn when added as the main carbohydrate source in extruded diets. Three diets containing 30% corn, 30% WSH, or 30% RSH were formulated to meet or exceed the AAFCO (2018) nutrient profiles for cats during growth. Nine male cats (0.8 ± 0.00 yr) were randomly assigned to one of the three dietary treatments using a triplicated 3 × 3 Latin square design. Experimental periods consisted of 14 d (10 d of diet adaption and 4 d of total and fresh fecal collections). The ATTD of dry matter (**DM**) did not differ amongst treatments, organic matter was greatest (*P* < 0.05) for both sorghum diets (86.4%) and lowest for the corn diet (84.2%), crude protein was comparable among diets ranging from 84.5 to 86.6%, acid hydrolyzed fat was high among diets varying between 91.4 and 92.8%, and total dietary fiber was greatest (*P* < 0.05) for the WSH diet (56.0%) with the corn diet being lowest (44.7%). Digestible energy was greatest (*P* < 0.05) for the WSH diet (4.66 kcal/g) and lowest for the corn diet (4.54 kcal/g), with the RSH diet being intermediate (4.64; *P* > 0.05). Fecal pH (6.3–6.5) and most fecal metabolites did not differ among diets except for phenol/indole concentrations that were significantly lower (*P* < 0.05) in cats fed the RSH diet (1.5 μmole/g DM) than for cats fed the corn diet (2.1 μmole/g DM). *Bacteroidetes, Firmicutes, Fusobacteria*, and *Proteobacteria* were the major phyla observed in the microbiota of feces of cats fed the three experimental diets, with no differences seen amongst all treatments. Data indicate that dietary supplementation of these varieties of WSH and RSH as carbohydrate sources were well-tolerated by the cat.

## Introduction

In recent years, there has been a trend for the companion animal sector to emulate human food market trends (1). As such, human interest in ancient grains replacing modern carbohydrate sources has reached the pet food market, and there has been increased focus on corn and wheat-free diets in pet foods. Due to the fact that sorghum (*Sorghum bicolor)* is considered an ancient grain (2), and the United States is the largest producer of this crop (3), sorghums can be an effective substitute for more traditional carbohydrate sources (i.e., rice and corn) in companion animal nutrition.

In some regions, rice (a common carbohydrate source in pet foods) can be more costly than sorghum (4). Since carbohydrates can be included in diets at concentrations of 30–60% on a DM basis (5), formulation costs can be substantial. In the U.S., sorghum has been used as an alternative ingredient in gluten-free products to replace wheat for human consumption (6). The chemical composition of the sorghum grain on a dry matter basis (**DMB**) is similar among different varieties, being comprised mainly of starch (~75%), protein (~12%), lipids (~4%), crude fiber (~3%), and ash [~ 2%; (7)]. The phytate and tannin concentrations in sorghum, however, can potentially lead to inconsistent utilization and digestibility of this cereal grain, which has been documented when sorghum based-diets were fed to broilers (8). In contrast, others (4, 5, 9) found their varieties of sorghum to be an acceptable carbohydrate source for canines and felines. Based on these previous studies, diets containing 55.2 and 59.3% sorghum, respectively, resulted in no detrimental effects on nutrient digestibility or fecal score, and resulted in a lower post-prandial insulin response compared with dogs fed diets containing similar amounts of rice (52.1 and 45.66%, respectively) or corn [53.5%; (10, 11)]. Additionally, the sorghum diet resulted in a lower blood glucose concentration compared with a corn diet, but with a similar insulin response for both (9).

Currently, little information is available regarding the effects of different varieties of sorghum as carbohydrate sources in pet foods and their impact on fecal fermentative end-products and microbiota of cats. Thus, the objective of this research was to evaluate the effect of dietary supplementation of red or white sorghum varieties in extruded diets on the gastrointestinal health of cats by determining apparent total tract macronutrient digestibility (**ATTD**), fecal characteristics and fermentative end-products, and fecal microbiota when compared with a corn-based extruded diet. Our hypothesis was that these varieties of red sorghum and white sorghum would be well-accepted by cats, and the red sorghum (**RSH**) and white sorghum (**WSH**) diets would be comparable to corn when added as the main carbohydrate source in extruded diets for cats.

## Materials and Methods

### Animals and Experimental Design

All animal procedures were approved by the University of Illinois Institutional Animal Care and Use committee (Protocol No. 17241). Nine, intact, male domestic shorthaired cats (average age: 0.8 ± 0.00 yr; average weight: 4.5 ± 0.23 kg) were used in a triplicated 3 × 3 Latin square design. Each period consisted of 10 d of diet adaptation and 4 d of total fecal collection. Cats were housed in a temperature-controlled room in the Edward R. Madigan Laboratory at the University of Illinois at Urbana-Champaign. The room was kept on a 14 h light/10 h dark schedule. Cats were group-housed except during meal times and for the duration of fecal collection periods. Cats were randomly assigned to one of the three experimental diets and were fed to maintain body weight (**BW**) and current body condition score of 5 using a 9-point scale, which were measured weekly during each experimental period (12). Water was available *ad libitum* and feeding was done twice daily at 800 and 1,500. Cats had access to their assigned food for 2 h (800–1,000 and 1,500–1,700) when food refusals, if present, were collected and recorded. During the collection phase, cats were housed individually in cages (73.7 cm long × 152.4 cm wide), given the same access to food and water, and had visual contact with the majority of cats in the room. During collection phases, cats were allowed access to litter-free collection boxes. The morning following the 4 d of total fecal and urine collections, blood draws were conducted for complete blood count (**CBC**) and serum metabolite analyses to assess health of the cats.

### Diets

Three diets containing 30% corn, 30% **WSH**, or 30% **RSH** were formulated to meet or exceed the Association of American Feed Control Officials [**AAFCO**; (13)] nutrient profiles for cats during growth. The diets were manufactured at the Grain Science and Industry Bioprocessing and Industrial Value-Added Products Innovation Center (BIVAP) at Kansas State University, Manhattan using a pilot-scale screw extruder (X-20; Wenger Manufacturing, Sabetha, KS, USA). All three experimental diets had similar ingredient composition except for the carbohydrate source (Table 1). Feed refusals were recorded after each meal throughout the study. Metabolizable energy requirements (100 kcal/kg BW^0.67^) were used to calculate food allowance for each individual cat to maintain BW and ideal body condition score. When necessary, food intake was adjusted accordingly.

**Table 1:** Ingredient composition of treatments containing different carbohydrate sources for adult cats.

**Ingredient, % as-fed basis**	**Treatments**^**a**^		
	**Corn**	**WSH**	**RSH**
Corn	30.00	–	–
White sorghum^b^	–	30.00	–
Red sorghum^b^	–	–	30.00
Poultry by-product meal	56.83	56.83	56.83
Corn gluten meal	6.09	6.09	6.09
Beet pulp	4.06	4.06	4.06
Sodium chloride	0.91	0.91	0.91
Potassium chloride	0.71	0.71	0.71
Choline chloride	0.51	0.51	0.51
Mineral premix^c^	0.30	0.30	0.30
Vitamin premix^c^	0.30	0.30	0.30
Myco-curb^d^	0.20	0.20	0.20
Naturox plus^d^	0.08	0.08	0.08

a *WSH, white sorghum; RSH, red sorghum*.

b *United Sorghum Checkoff Program*.

c *Minerals provided per kg diet: 17.4 mg manganese (MnSO_4_), 284.3 mg iron (FeSO_4_), 17.2 mg copper (CuSO_4_), 2.2 mg cobalt (CoSO_4_), 166.3 mg zinc (ZnSO_4_), 7.5 mg iodine (KI), and 0.2 mg selenium (Na_2_SeO_3_). Vitamins provided per kg diet: 11,000 IU vitamin A Acetate; 900 IU vitamin D3; 57.5 IU vitamin E Acetate; 0.6 mg vitamin K; 7.6 mg thiamine HCl; 11.9 mg riboflavin; 18.5 mg pantothenic acid; 93.2 mg niacin; 6.6 mg pyridoxine HCl; 12.4 mg biotin; 1,142.1 μg folic acid; 164.9 μg vitamin B12, 0.1% mannitol*.

d *Myco-Curb = mold inhibitor (Kemin; Des Moines, IA); Naturox Plus = mixed-tocopherol antioxidant (Kemin; Des Moines, IA)*.

### Sample Collection

Throughout the 4 d of total fecal collection, all feces were collected, weighed, and scored using the following 5-point scale: 1= hard, dry pellets; small hard mass; 2 = hard formed, remains firm and soft; 3 = soft, formed and moist stool, retains shape; 4 = soft, unformed stool; assumes shape of container; 5 = watery, liquid that can be poured. All individual fecal samples identified by cat and period were stored in a −20°C freezer until chemical analyses were conducted and **ATTD** of macronutrients was determined.

One fresh fecal sample from each cat was collected within 15 min of defecation and analyzed for dry matter (**DM**), phenols and indoles, short-chain fatty acids (**SCFA**), branched-chain fatty acids (**BCFA**), and ammonia. The pH, fecal score, and total sample weight also were determined. Dry matter was measured by drying ~2 g of feces in duplicate in a 105°C forced air oven until all moisture was removed (48 h). Approximately 2 g of feces in duplicate were stored in plastic tubes covered in Parafilm and frozen at −20°C for subsequent indole and phenol analyses. Four grams of sample were stored in Nalgene bottles containing 4 mL of 2N hydrochloric acid and frozen at −20°C to determine SCFA, BCFA, and ammonia concentrations. Fecal samples allocated for microbiota were stored in 2 mL cryovials and stored at −80°C until analysis.

After overnight fasting, 4 mL of blood were collected via jugular venipuncture from each cat at the end of each experimental period. All cats were sedated prior to collection using 0.9 ml/kg of a mix of Dexmedetomidine (0.062 mg/mL), Ketamine (62.4 mg/mL), and Butorphanol (2.5 mg/mL). Sedation was reversed by giving 0.1 mL of Atipemezole (5 mg/mL) to each cat. Blood samples from each cat were collected in BD Vacutainer serum separator tubes and EDTA tubes (Becton, Dickinson and Company, Franklin Lakes, NJ) that were used for serum metabolite and complete blood count analyses, respectively. These analyses were conducted by the Clinical Pathology Laboratory at the University of Illinois College of Veterinary Medicine (Urbana, IL).

### Chemical Analyses

Fecal samples from each cat and period were pooled and dried in a 57°C oven before grinding in a Wiley mill (model 4, Thomas Scientific, Swedesboro, NJ) with a 10 mesh (2 mm) screen size and used for subsequent analyses. Dry matter, organic matter (**OM**), and ash were determined for the diets and feces using the Association of Official Analytical Chemists (AOAC) procedure [(14); methods 934.01 and 942.05]. Acid-hydrolyzed fat (**AHF**) of the diet and feces were conducted following methods of the American Association of Cereal Chemists [(15); method 30–14] and Budde et al. (16). Crude protein (**CP**) analysis was conducted by measuring total nitrogen using a LECO TruMac (Leco Corporation, St. Joseph, MI; model 630-300-300) and following the Official Method of AOAC International [(14); method 992.15]. Gross energy (**GE**) of diets and feces were measured using a Parr 6200 calorimeter (Parr Instrument Company, Moline, IL). Total dietary fiber (**TDF**) was analyzed according to Prosky et al. (17) and the Official Method of AOAC International [(14); Methods 985.29 and 991.43].

Short-chain fatty acids and BCFA concentrations were analyzed using gas chromatography with a glass 6'x1/4” ODx4mmID column and 10%SP1200/1%H_3_PO_4_ on 80/100 Chrom-WAW, Supleco packing and following the methods of Erwin et al. (18), Supleco Inc. (19), and Goodall and Byers (20). Gas chromatography also was used to measure phenols and indoles as cited in Flickinger et al. (21). Fecal ammonia concentrations were determined using gas chromatography according to Chaney and Marbach (22).

### DNA Extraction, Amplification, Sequencing, and Bioinformatics

Total DNA from fresh fecal samples was extracted using Mo-Bio PowerSoil kits (MO BIO Laboratories, Inc., Carlsbad, CA) and DNA concentration was quantified using a Qubit® 3.0 Fluorometer (Life technologies, Grand Island, NY). Amplification of the 16S rRNA gene was completed using a Fluidigm Access Array (Fluidigm Corporation, South San Francisco, CA) in combination with Roche High Fidelity Fast Start Kit (Roche, Indianapolis, IN). The primers 515F (5′-GTGCCAGCMGCCGCGGTAA-3′) and 806R (5′-GGACTACHVGGGTWTCTAAT-3′) that target a 252 bp-fragment of V4 region was used for amplification [primers synthesized by IDT Corp., Coralville, IA; (23)]. Fluidigm specific primer forward (CS1) and reverse (CS2) tags was added according to the Fluidigm protocol. Fragment Analyzer (Advanced Analytics, Ames, IA) was used to confirm the quality of amplicons' regions and sizes. A DNA pool was generated by combining equimolar amounts of the amplicons from each sample. The pooled samples were sized selected on a 2% agarose E-gel (Life technologies, Grand Island, NY) and extracted using Qiagen gel purification kit (Qiagen, Valencia, CA). Cleaned size-selected pooled products were run on an Agilent Bioanalyzer to confirm appropriate profile and average size. Illumina sequencing was performed on a MiSeq using v3 reagents (Illumina Inc., San Diego, CA) at the W. M. Keck Center for Biotechnology at the University of Illinois. Fluidigm tags were removed using FASTX-Toolkit (version 0.0.14), and sequences were analyzed using QIIME 2.0, version 2020.6 (24) and DADA2 [version 1.14; (25)]. High quality (quality value ≥ 20) sequence data derived from the sequencing process were demultiplexed. Sequences then were clustered into operational taxonomic units (OTU) using opened-reference OTU picking against the SILVA 138 reference OTU database with a 97% similarity threshold (26). Singletons (OTUs that are observed fewer than two times) and OTUs that had <0.01% of the total observation were discarded. A total of 949,169 reads were obtained, with an average of 58,392 reads (range = 31,047–44,759) per sample. The dataset was rarified to 36,130 reads for analysis of diversity and species richness. Principal coordinates analysis (**PCoA**) was performed, using both weighted and unweighted unique fraction metric (**UniFrac**) distances that measures the phylogenetic distance between sets of taxa in a phylogenetic tree as the fraction of the branch length of the tree, on the 97% OTU composition and abundance matrix (27).

### Statistical Analysis

Data were analyzed using SAS (SAS Institute, INC., version 9.4, Cary, NC) using MIXED model procedures. The statistical model included the fixed effect of diet and the random effect of animal with each cat being the experimental unit. Data normality (based on residuals) was checked using the UNIVARIATE procedure of SAS. All treatment least-squares means were compared with each other and Tukey adjustment was used to control for the Type 1 experiment-wise error. *P*-values < 0.05 were considered statistically different, Standard errors of the mean (**SEM**) were reported as determined from the MIXED models procedure of SAS.

## Results

### Composition of Raw Grains

Overall, the proximate analyses of the raw corn, WSH, and RSH were comparable (Table 2). Dry matter content ranged from 88.3 to 89.6%, OM ranged from 98.3 to 98.6%, CP ranged from 9.1 to 11.5%, AHF ranged from 3.6 to 4.9%, and GE ranged from 4.38 to 4.45 kcal/g. The TDF concentration for the raw corn, WSH, and RSH ranged from 12.5 to 15.9%. The insoluble fiber concentration varied from 9.4 to 13.1%, whereas the soluble fiber concentration fluctuated from 2.8 to 3.1%. The primary difference among grain sources was that the RSH contained more CP, TDF, and insoluble fiber than the corn and WSH.

**Table 2:** Analyzed chemical composition of raw grains (DMB)^a^.

**Proximate analysis**	**Ingredient**^**a**^		
	**Raw corn**	**Raw WSH**	**Raw RSH**
Dry matter (%)	88.4	88.3	89.6
Organic matter (%)	98.6	98.5	98.3
Ash (%)	1.4	1.5	1.7
Crude protein (%)	9.1	9.4	11.5
Acid hydrolyzed fat (%)	4.9	3.6	4.3
Gross energy (kcal/g)	4.39	4.38	4.45
Total dietary fiber (%)	13.9	12.5	15.9
Insoluble fiber (%)	11.0	9.4	13.1
Soluble fiber (%)	2.8	3.1	2.8
Starch (%)	62.7	66.4	59.2
Trypsin inhibitor (TIU/g)	751.0	n/d	514
Phytic acid (%)	1.0	1.1	1.1
Protein dispersibility index (%)^b^	45.8	16.9	14.6
Tannins (%)	0.1	0.1	0.2

a *DMB, dry matter basis; WSH, white sorghum; RSH, red sorghum*.

b *Protein dispersibility index is a means of comparing the solubility of protein in water, with a greater value indicating more solubility*.

### Food Intake, Apparent Total Tract Digestibility of Macronutrients, and Fecal Characteristics

All three diets were formulated targeting a similar nutrient profile and to be isonitrogenous and isocaloric (Table 1). Analyzed chemical composition of the experimental diets revealed that all diets had similar nutrient composition (Table 3).

**Table 3:** Analyzed chemical composition of diets containing different carbohydrate sources for cats.

**Item**	**Treatments**^**a**^		
	**Corn**	**WSH**	**RSH**
Dry matter, %	94.2	94.1	94.2
	**% DM**^**b**^ **basis**		
Organic matter	89.7	89.9	89.5
Ash	10.3	10.1	10.6
Acid hydrolyzed fat	18.0	18.9	18.1
Crude protein	45.0	44.7	44.2
Total dietary fiber	13.3	14.2	13.6
Soluble dietary fiber	4.1	5.7	4.7
Insoluble dietary fiber	9.2	8.5	8.9
Gross energy, kcal/g	5.34	5.36	5.32
**Amino acids, % as-fed basis**	**Treatments**^**a**^		
	**Corn**	**WSH**	**RSH**
**Essential**			
Arginine	2.7	2.7	2.5
Histidine	0.9	0.9	0.9
Isoleucine	1.8	1.8	1.7
Leucine	3.5	3.6	3.5
Lysine	2.5	2.5	2.4
Methionine	0.8	0.8	0.8
Phenylalanine	1.9	1.9	1.8
Threonine	1.6	1.6	1.6
Tryptophan	0.4	0.4	0.4
Valine	2.1	2.1	2.0
Taurine	0.4	0.4	0.4
**Non-essential**			
Alanine	2.9	2.9	2.9
Aspartate	3.4	3.4	3.2
Cysteine	0.5	0.5	0.5
Glutamate	5.7	5.9	5.7
Glycine	3.6	3.4	3.4
Proline	2.8	2.7	2.7
Serine	1.6	1.6	1.5
Tyrosine	1.4	1.5	1.4

a *WSH, white sorghum; RSH, red sorghum*.

b *DM, dry matter*.

Daily food intake (DMB), fecal output g/d (as-is), and fecal score did not differ among treatments (Table 4). However, fecal output (g/d; DMB) for cats fed the RSH diet was lower (*P* < 0.05) than for the corn diet, with the WSH diet being intermediate (*P* > 0.05; Table 4). The ATTD of DM, CP, and AHF were not affected by treatment. Organic matter and TDF digestibility for cats fed WSH and RSH diets were not different from one another, but were greater (*P* < 0.05) than for the corn diet. Digestible energy of the WSH diet was higher (*P* < 0.05) than for corn, with RSH being intermediate (*P* > 0.05).

**Table 4:** Body weight, food intake, fecal characteristics, and total tract apparent macronutrient digestibility of cats fed diets containing different carbohydrate sources.

**Item**	**Treatments**^**a**^			**SEM^**b**^**
	**Corn**	**WSH**	**RSH**	
Body weight (kg)	4.7	4.7	4.7	0.17
Food intake, g/d (DMB)	74.3	73.9	74.1	0.92
Fecal output, g/d (as-is)	44.5	40.6	40.7	2.11
Fecal output, g/d (DMB)	16.1^c^	14.0^cd^	14.0^c^	0.62
Fecal score	2.4	2.5	2.3	0.09
Digestibility, %				
Dry matter	78.3	81.0	81.1	0.88
	**% DM**^**a**^ **basis**			
Organic matter	84.2^d^	86.4^c^	86.4^c^	0.67
Acid hydrolyzed fat	91.4	92.8	92.8	0.48
Crude protein	84.5	86.0	86.6	0.73
Total dietary fiber	44.7^d^	56.0^c^	53.2^c^	2.25
Digestible energy, kcal/g	4.54^d^	4.66^c^	4.64^cd^	0.03

a *DMB, dry matter basis, DM, dry matter; WSH, white sorghum; RSH, red sorghum*.

b *Standard error of the mean*.

cd *Means within a row with no common superscript letter are different (P < 0.05)*.

Fecal pH and concentrations of ammonia (μmole/g DMB) did not differ among treatments, while phenol concentrations were not detected in sufficient quantities among treatments (Table 5). Similarly, fecal concentrations of SCFA and BCFA did not differ among treatments. However, total phenols/indoles and indoles alone were higher (*P* < 0.05) for the corn treatment compared with the RSH treatment, the WSH diet was intermediate (*P* > 0.05; Table 5).

**Table 5:** Fecal fermentative-end products for cats fed diets containing different carbohydrate sources.

**Item, μmole/g DM^**a**^**	**Treatments**^**b**^			**SEM^**c**^**
	**Corn**	**WSH**	**RSH**	
Fecal pH	6.5	6.3	6.3	0.13
Ammonia	114.6	127.4	129.1	8.46
Total phenols/indoles	2.1^e^	1.9^ef^	1.5^f^	0.14
Phenols^d^	ND	ND	ND	–
Indoles	2.1^e^	1.9^ef^	1.5^f^	0.13
Total short-chain fatty acids	364.0	359.3	404.0	35.32
Acetate	236.6	231.6	263.4	25.31
Propionate	89.8	82.9	94.8	8.22
Butyrate	37.7	44.8	45.8	3.56
Total branched-chain fatty acids	25.8	27.9	30.1	2.35
Isobutyrate	5.9	5.8	6.5	0.44
Isovalerate	8.3	8.6	9.2	0.68
Valerate	11.6	13.5	14.4	1.62

a *DM, dry matter, all values except fecal pH expressed as μmole/g DM*.

b *WSH, white sorghum; RSH, red sorghum*.

c *Standard error of the mean*.

d *Not detected*.

ef *Means within a row with no common superscript letter are different (P < 0.05)*.

Serum metabolite profiles of cats fed diets containing WSH, RSH, or corn were mostly within reference ranges for healthy cats and did not differ among treatments, except for calcium which was highest (*P* < 0.05) for WSH and lowest for RSH, corn was intermediate, but still was within reference range (Table 6). Metabolites in excess of the reference range (i.e., glucose and phosphorus) might be due to the administration of the sedative, and were not affected by treatment. Likewise, CBC results were normal among all cats and did not differ among dietary treatments (data not shown).

**Table 6:** Fasted serum metabolite profiles for cats fed diets containing different carbohydrate sources.

**Item**	**Reference range**	**Treatments**^**a**^			**SEM^**b**^**
		**Corn**	**WSH**	**RSH**	
Creatinine, mg/dL	0.4–1.6	1.41	1.50	1.50	0.069
Blood urea nitrogen, mg/dL	18–38	23.56	24.44	24.11	0.809
Total protein, g/dL	5.8–8.0	6.24	6.36	6.23	0.091
Albumin, g/dL	2.8–4.1	3.34	3.38	3.37	0.045
Globulin, g/dL	2.6–5.1	2.90	2.98	2.87	0.082
Albumin/globulin, g/dL	0.6–1.1	1.16	1.13	1.18	0.042
Calcium, mg/dL	8.8–10.2	9.30^cd^	9.44^c^	9.24^d^	0.075
Phosphorus, mg/dL	3.2–5.3	5.81	5.90	5.83	0.149
Sodium, mmol/L	145–157	149.00	149.22	149.56	0.332
Potassium, mmol/L	3.6–5.3	4.32	4.32	4.27	0.085
Sodium/potassium ratio	28–36	34.56	34.67	35.22	0.719
Chloride, mmol/L	109–126	115.33	115.22	115.56	0.440
Glucose, mg/dL	60–122	143.00	133.78	146.67	10.448
Alkaline phosphatase total, U/L	10–85	52.78	57.00	53.56	3.770
Alanine aminotransferase, U/L	14–71	47.44	49.22	49.56	4.036
Gamma-glutamyl transferase, U/L	0–3	0.33	0.00	0.22	0.128
Total bilirubin, mg/dL	0.0–0.3	0.11	0.11	0.11	0.011
Creatine kinase, U/L	10–250	233.11	311.22	321.67	37.279
Cholesterol total, mg/dL	66–160	129.44	134.56	117.67	10.286
Triglycerides, mg/dL	21–166	38.33	40.22	37.00	3.720
Bicarbonate (TCO_2_), mmol/L	12–21	19.33	19.11	19.33	0.311
Anion gap	10–27	18.56	19.22	18.89	0.539

a *WSH, white sorghum; RSH, red sorghum*.

b *Standard error of the mean*.

cd *Means within a row with no common superscript letter are different (P < 0.05)*.

### Fecal Microbial Populations

**Figure 1:**
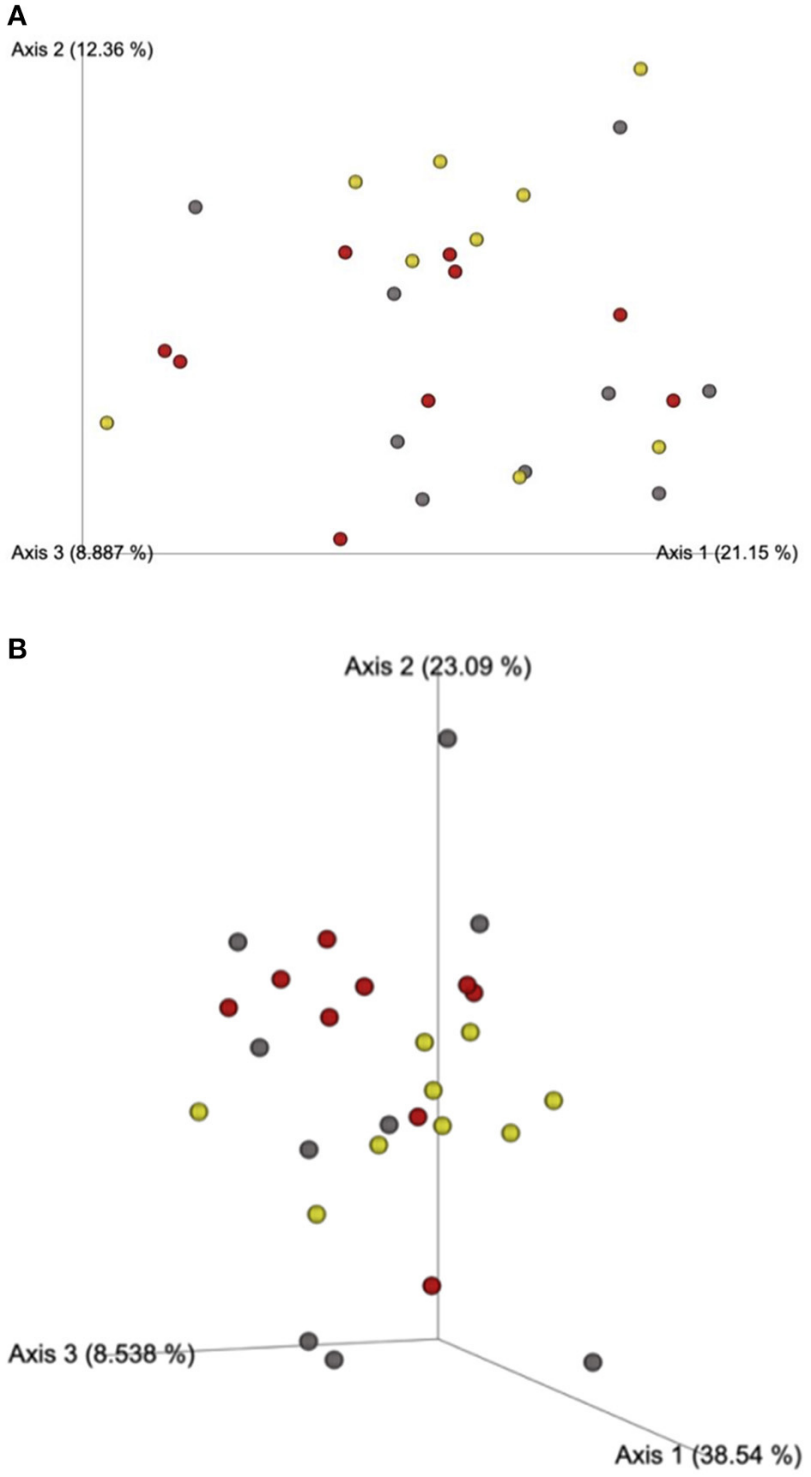
**(A)** Fecal microbial β-diversity based on unweighted* UNIFRAC analysis of cats fed diets containing different carbohydrate sources. Yellow dots = corn diet (control), red dots = red sorghum diet, gray dots = white sorghum diet. *Unweighted considers the presence or absence of observed taxa incorporating phylogenetic distances, with each dot representing a cat. **(B)** Fecal microbial β-diversity based on weighted* UNIFRAC analysis for cats fed diets containing different carbohydrate sources. *Weighted considers the proportion of observed taxa incorporating phylogenetic distances (the relative relatedness of microbial community), with each dot representing a cat.

**Figure 2:**
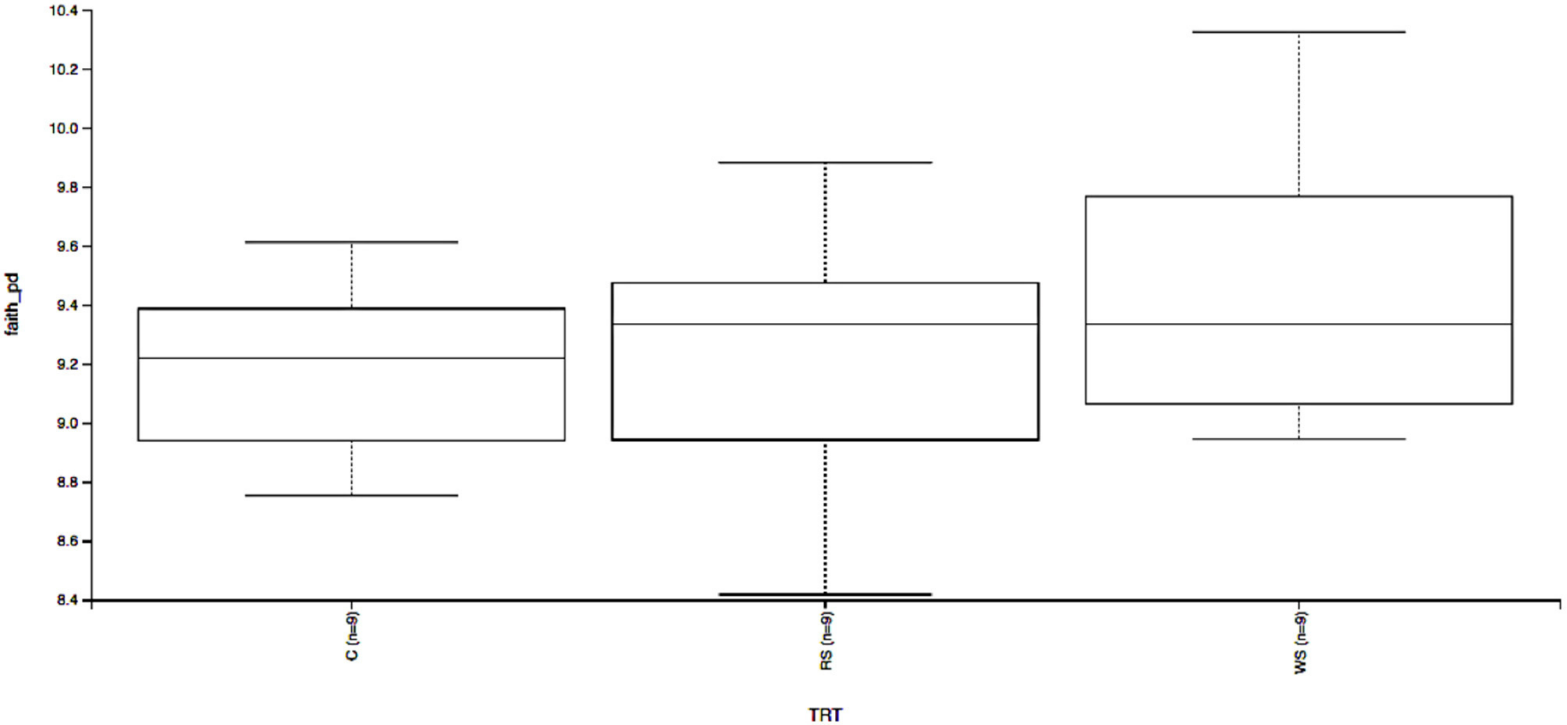
Fecal microbial α-diversity* based on Faith's phylogenetic diversity of cats fed diets containing different carbohydrate sources. C = corn diet (control), RS = red sorghum diet, WS = white sorghum diet, TRT = treatment, Y-axis is Faith's phylogenetic diversity, X-axis left to right C, RS, WS (*n* = 9). * α-diversity measures species richness within sample, and whole trees indicate it was not affected by treatment (*P* > 0.05).

**Figure 3:**
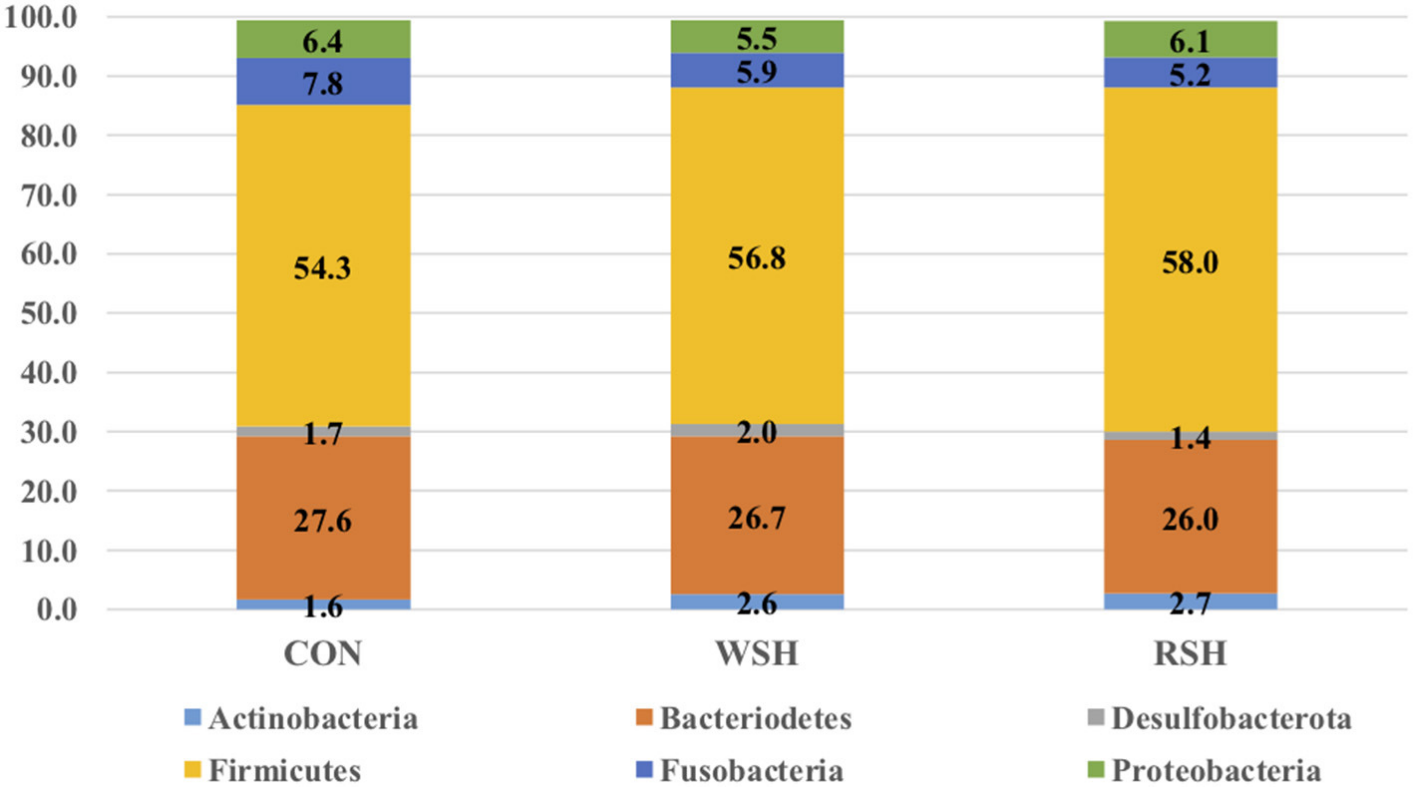
Fecal microbial relative abundance (%, total sequences) of predominant phyla of cats fed diets containing different carbohydrate sources. CON = corn diet (control), WSH = white sorghum diet, RSH = red sorghum diet.

Beta-diversity based on unweighted (Figure 1A) and weighted (Figure 1B) UniFrac distances and alpha-diversity did not differ in feces of cats fed corn, WSH, or RSH (Figure 2). A total of six bacterial phyla were observed with Firmicutes, Bacteroidetes, Fusobacteria, and Proteobacteria being the most predominant phyla, comprising more than 90% of all sequences (Figure 3).

**Figure 4:**
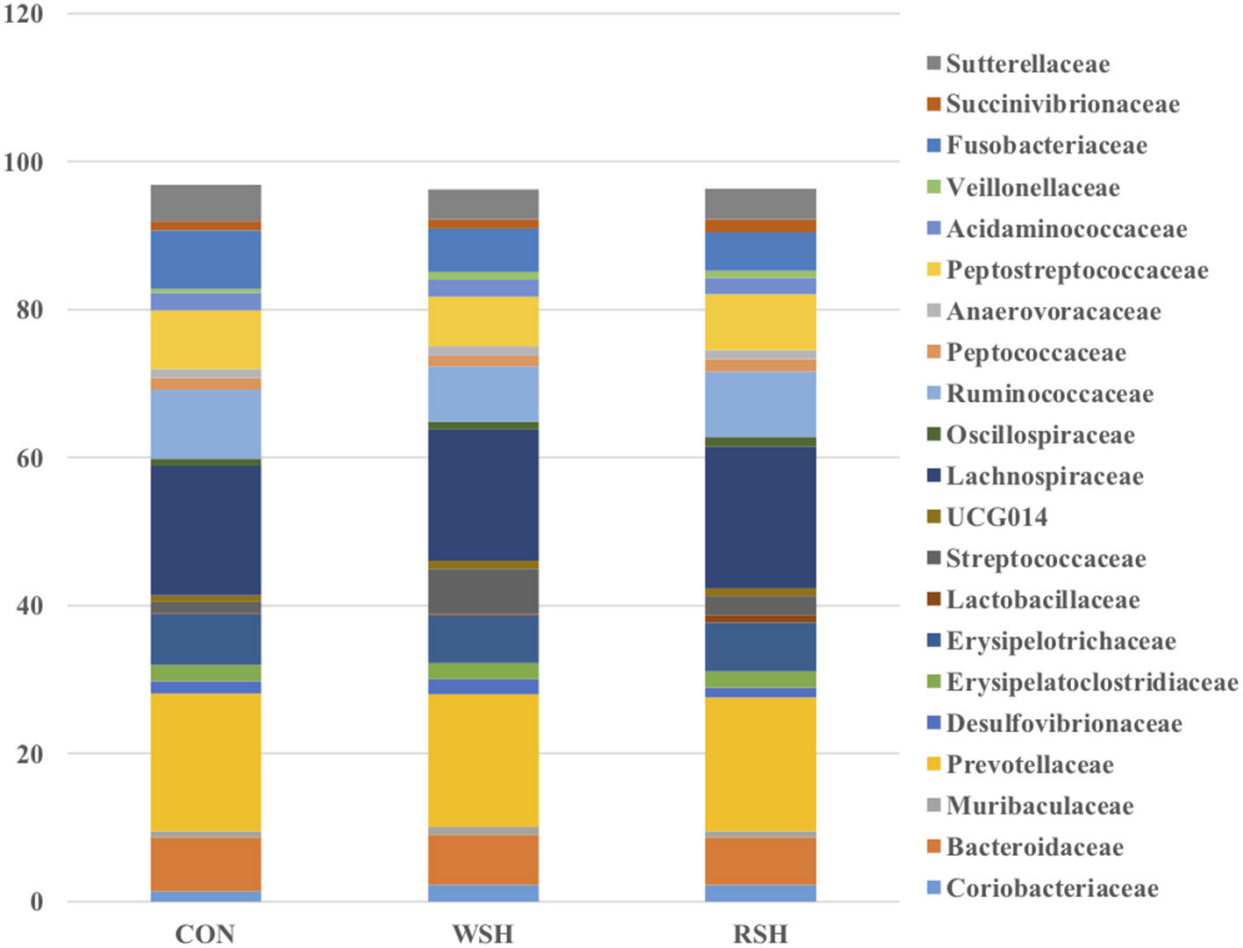
Family fecal microbial relative abundance (%, total sequences) of cats fed diets containing different carbohydrate sources. CON = corn diet (control), WSH = white sorghum diet, RSH = red sorghum diet.

**Figure 5:**
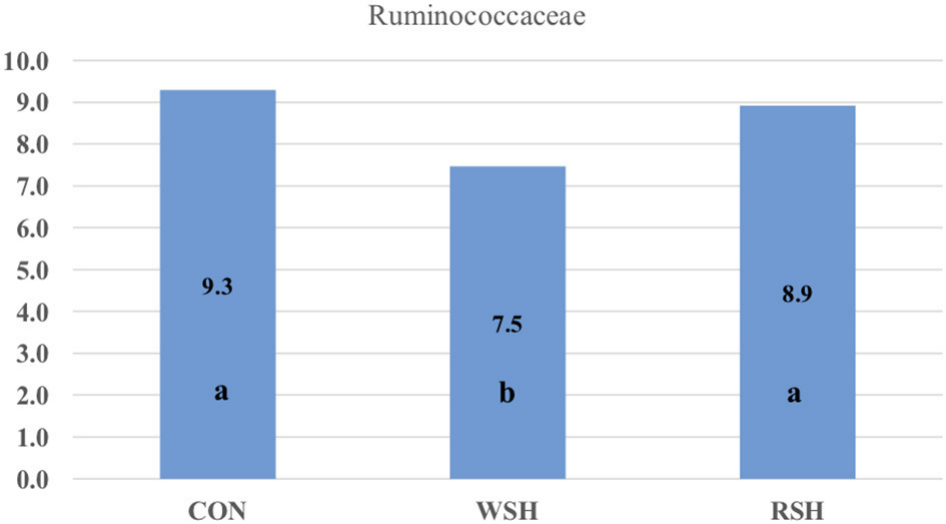
Significant bacterial family group in feces of cats fed diets containing different carbohydrate sources. CON = corn diet (control), WSH = white sorghum diet, RSH = red sorghum diet. ^ab^Bars containing no common letters **(a,b)** represent statistical difference (*P* < 0.05).

Twenty-one bacterial families were observed, accounting for ~96% of all sequences. The most predominant taxa were *Prevotellaceae* (Bacteroidetes), *Lachnospiraceae* (Firmicutes), *Ruminococcaceae* (Firmicutes), *Peptostreptococcaceae* (Firmicutes), *Bacteroidaceae* (Bacteroidetes), *Erysiplotrichaceae* (Firmicutes), and *Fusobacteriaceae* (Fusobacteria), accounting for ~70% of all sequences (Figure 4). Only *Ruminococcaceae* (Firmicutes) was different (*P* < 0.05) amongst the three dietary treatments (Figure 5). Cats fed the corn (9.3%) and RSH (8.9%) diets had greater (*P* < 0.05) relative abundance of *Ruminococcaceae* compared with cats fed the WSH diet (7.5%).

## Discussion

### Grain Composition

The compositions of the raw grains were comparable and agree well with previous literature (7, 28–31). The current study corn and sorghum values were in strong agreement with the values determined by the Bazolli et al. (32) study who determined values on a DMB for their maize and sorghum of 88.3 and 88.8% DM, 1.2 and 1.4% ash, 9.1 and 10.2% CP, 6.3 and 3.8% fat, and 11.2 and 12.6% TDF, respectively.

Minor differences were determined amongst the three grains. Of the three grains, the raw RSH was ~2 percentage units greater in CP than either the raw corn or the raw WSH. Additionally, raw RSH was ~2–3% units greater in TDF than either of the other two grains. The increase in CP for RSH was expected as RSH traditionally has more CP compared with white varieties. The current study CP values for RSH and WSH agree with previous research that reported values of 13.4 and 9.8% for red and white sorghum, respectively (33). The TDF concentrations of the RSH and WSH were analyzed in the current study while Selle et al. (33) measured crude fiber content. While a direct comparison among these 2 types of fiber methods is inappropriate, both the current study and Selle et al. (33) reported greater fiber content for red sorghum compared with white. The greater TDF concentration for RSH compared with the other two grains was expected due to the more comprehensive characterization of the fiber content of RSH varieties.

### Apparent Total Tract Digestibility

The sorghum diets in the current study had greater ATTD values compared with de-Oliveira et al. (9) study which determined ATTD values for their sorghum diet when fed to cats of 76.3% for DM, 80.0% for OM, 80.6% for CP, and 83.3% for AHF. The ATTD of the corn diet in the current study was comparable to the values determined in the de-Oliveira et al. (9) study for their corn diet values of 78.5% for DM, 82.5% for OM, 83.2% for CP. The current study determined higher ATTD of TDF for the corn and both sorghum diets compared with de-Oliveira et al. (9). Contrary to current study, de-Oliviera et al. found that the TDF digestibility was not different than a corn diet (9).

Information about feline digestibility of energy and nutrients is scarce, for that reason comparisons of other feline diets incorporating different carbohydrate sources are warranted. The current study found comparable results to Thiess et al. (34) that fed a high carbohydrate diet (40.3%) to male cats with ATTD values of 84.5% for DM, 86.0% for OM, 82.0% for CP, and 95.3% for crude fat. The carbohydrate source in Thiess et al. (34) was steamed polenta, and its ATTD agreed well with the ATTD of the corn diet used in the current study, as well as both sorghum diets. While the fat extraction method varied from the current study, it is still applicable to showing that high fat (>90%) ATTD is observed in feline nutrition.

Asaro et al. (35) determined the ATTD in three different commercial diets with ingredients that had different glycemic responses. The medium glycemic response diet contained corn and whole grain sorghum as the carbohydrate sources. The ATTD of the diet was similar to the current study diets with values of 86.2% for DM, 89.5% for OM, 87.3% for CP, and 95.4% for fat. The 8-percentage unit increase in DM ATTD compared with the corn diet in the current study could be attributed to the inclusion of whole grain sorghum in the medium glycemic response diet, as the current study saw a significant increase in OM ATTD for both sorghum diets compared with corn. Asaro et al. (35) obtained a digestible energy (**DE**) concentration (4.75 kcal/g) that was similar to the current study values.

Barry et al. (36) fed a diet consisting of 27.8% brewer's rice and found ATTD values that were similar to the current study (81.4% for DM, 84.4% for OM, 84.2% for CP, and 95.7% for AHF). The ammonia level of 110.0 μmole/g DM agreed with the current study values regardless of dietary treatment. Both the pH (6.7) and fecal score (2.7) were elevated compared with the current study. Additionally, total SCFA values were smaller 246.7 μmole/g DM compared with the current study values (36). This could be due to the lower fiber content of brewer's rice and greater ATTD compared with corn and sorghum. The total BCFA concentration (31.3 μmole/g DM) from Barry et al. (36) agreed with the current study with the strongest agreement being observed with the WSH diet.

### Fecal Microbial Populations

The gastrointestinal microbiome, which is becoming recognized as a metabolically active organ, of cats has been increasingly recognized as being associated with pet health (37). The gastrointestinal tract of animals and humans is the primary microbial habitat and has a profound impact on their host (38). Previously, most microbiome research centered on the characterization of altered composition in diseased states; however, mounting evidence of dietary components impacting allergies, oral health, diabetes, and weight management through gut microbiome is becoming clearer (37). The age, sex, breed, amount, and form of food, health status, and composition of diet can all influence the gastrointestinal microbiome (38, 39).

The gastrointestinal microbiota of cats has been reported as similar to dogs (40), the dominant microbial phyla in both consisting of *Bacteroidetes, Firmicutes, Fusobacteria, Proteobacteria*, and *Actinobacteria* (38, 41). However, others have reported that the feline microbiota is more varied and diverse in composition, and this variation can be attested to adaption of the microbiome to different diets [omnivorous for canine, carnivorous for feline; (37)]. Kanakupt et al. (42) determined *Bacteroidetes* (36.1%), *Firmicutes* (36.3%), *Proteobacteria* (12.4%), and *Actinobacteria* (7.7%) to be dominate phyla in their feline fecal samples in adult cats fed an extruded diet containing rice and corn as the main carbohydrate sources. At 42 weeks of age (similar aged cats were used in the current study), the relative abundance of major phyla for felines consisted of 11.8% *Actinobacteria*, 34.7% *Bacteroidetes*, 36.1% *Firmicutes*, and 11.4% *Proteobacteria* (43). The most predominant phyla for 16-week-old cats fed a moderate carbohydrate (40.4% dried potato product) and moderate protein (30.8% chicken meal) extruded diet was *Firmicutes* [71.0%; (44)]. The predominant phyla reported by Kanakupt et al. (42), Deusch et al. (43), and Hooda et al. (44) are in agreement with the current study, *Bacteriodetes* (26.0−27.6%), *Firmicutes* (54.3−58.0%), and *Proteobacteria* (5.5−6.4%). However, the current study had greater levels of *Fusobacteria* (5.2−7.8%) compared with *Actinobacteria* (1.6 – 2.7%).

Ritchie et al. (45) found the major bacterial families in the feces consisted of *Bacteroidaceae, Porphyromonadaceae, Prevotellaceae*, and *Rikenellaceae* in cats fed an extruded commercial diet. *Bacteroidaceae, Eubacteriaceae, Clostridiaceae, Streptococcacceae*, and *Lactobacillaceae* have also been reported as dominant families in feline fecal samples by Itoh et al. (46) and Rochus et al. (47). In the present study, *Bacteroidaceae* and *Prevotellaceae* also were predominant families among cats fed corn, WSH, or RSH. However, other predominant families *Lachnospiraceae, Peptostreptococcaceae, Erysiplotrichaceae*, and *Fusobacteriaceae* were also prevalent and at a higher relative abundance compared with previous research. The current study relative abundance of *Ruminococcaceae* (7.5−9.3%) was in agreement with Hooda et al. (44) who determined a relative abundance of 7.16%. The increase (*P* < 0.05) in relative abundance for *Ruminococcaceae* for cats fed the corn and RSH diets compared with the WSH diet could be due to greater fiber and carbohydrate substrates reaching the hindgut of cats fed those diets. *Ruminococcaceae* are able to process a wide range of substrates from glucose and cellobiose to cellulose and hemicellulose (48). In humans, increase in fecal concentrations of SCFA-producing bacteria have been reported in response to increased intake of dietary fiber (49–51).

The differences between the current study and previous studies evaluating fecal microbiota of cats might be attributed to differences in diets being fed, breed of cats used, stage of life, environmental factors, and sequencing methods and bioinformatics analysis used. Overall, the relative abundance of fecal microbial taxonomic populations of cats fed corn, WSH, and RSH was not altered, which agrees with minor changes observed in fecal concentration of metabolites derived from microbial fermentation in the feline hindgut.

### Serum Biochemical Differences

The significant differences observed in the fasted serum calcium profile (Table 6). While there were significant differences among treatments, all fasted serum calcium concentrations were within the reference range and likely had no impact on the physiology and health of these animals.

## Conclusions

The data demonstrate that diets formulated with up to 30% of WSH or RSH are well-tolerated, had greater ATTD of OM and TDF, and resulting in no signs of gastrointestinal discomfort or intolerance when fed to cats. Overall, coefficients of digestibility for the macronutrients analyzed were high and comparable to extruded commercial feline diets. Fecal concentrations of SCFA (e.g., acetate, propionate, and butyrate) indicate that WSH and RSH diets resulted in comparable saccharolytic hindgut fermentation and fecal microbiota as the corn diet, whereas red sorghum variety also resulted in lower fecal indole concentration.

## Data Availability Statement

The data presented in the study are deposited in the Illinois Data Bank repository, accession number doi: 10.13012/B2IDB-2580847_V1.

## Ethics Statement

The animal study was reviewed and approved by University of Illinois Institutional Animal Care and Use Committee.

## Author Contributions

MRCG designed the experiment. PvS and FH performed the laboratory analyses. PvS and MRCG performed the statistical analyses. BRS and SLR-Z performed bioinformatic analysis for fecal microbial analysis. PvS wrote the manuscript. All authors revised and provided intellectual input on this manuscript.

## Conflict of Interest

The authors declare that the research was conducted in the absence of any commercial or financial relationships that could be construed as a potential conflict of interest.
